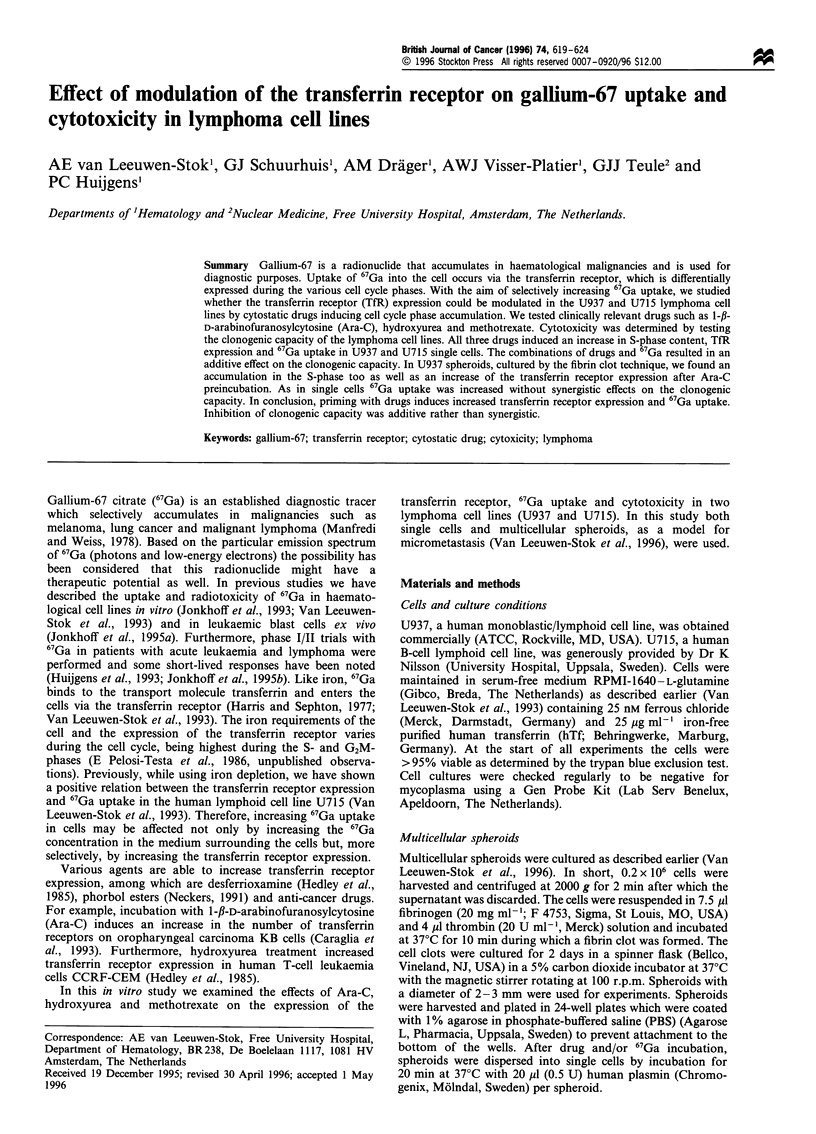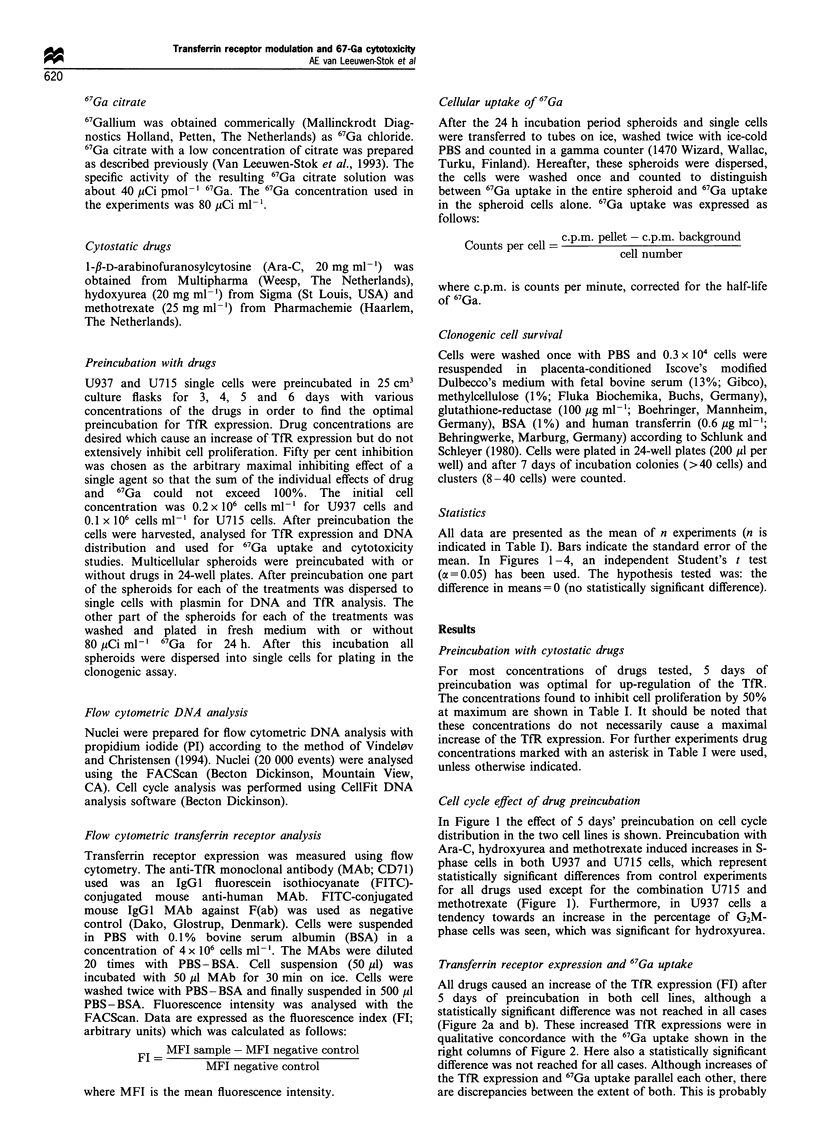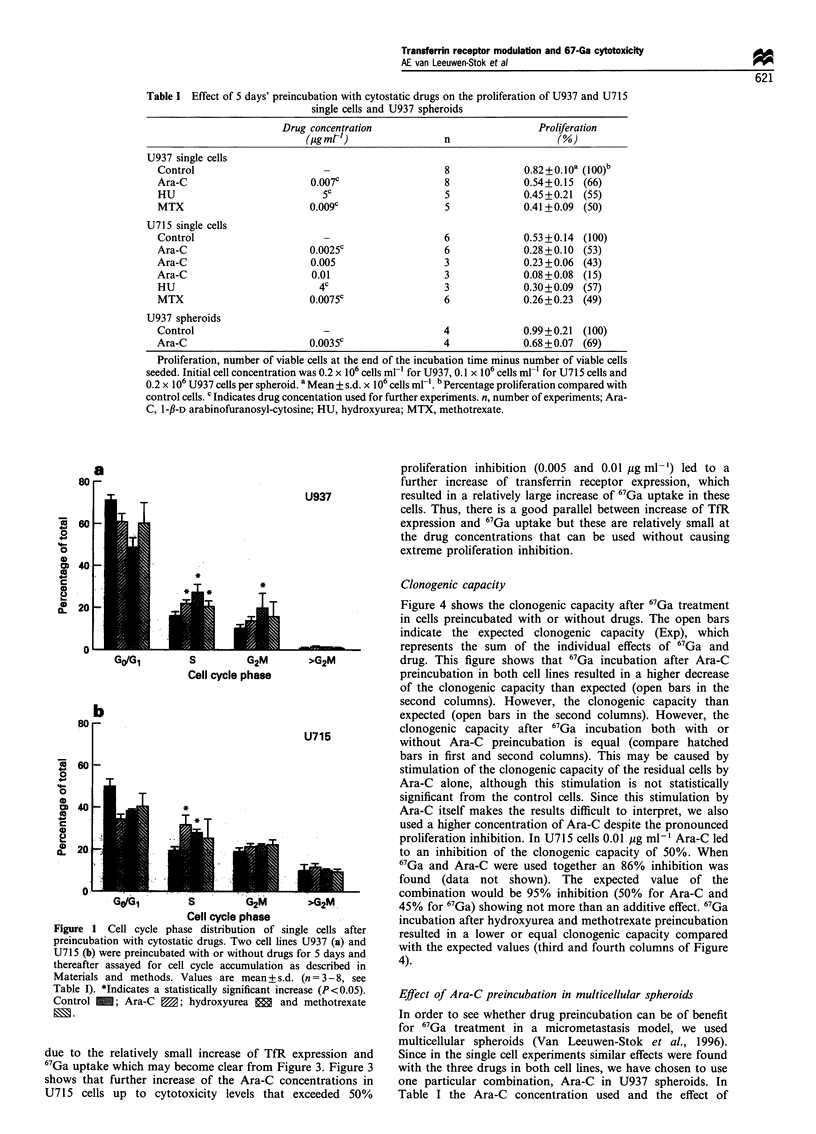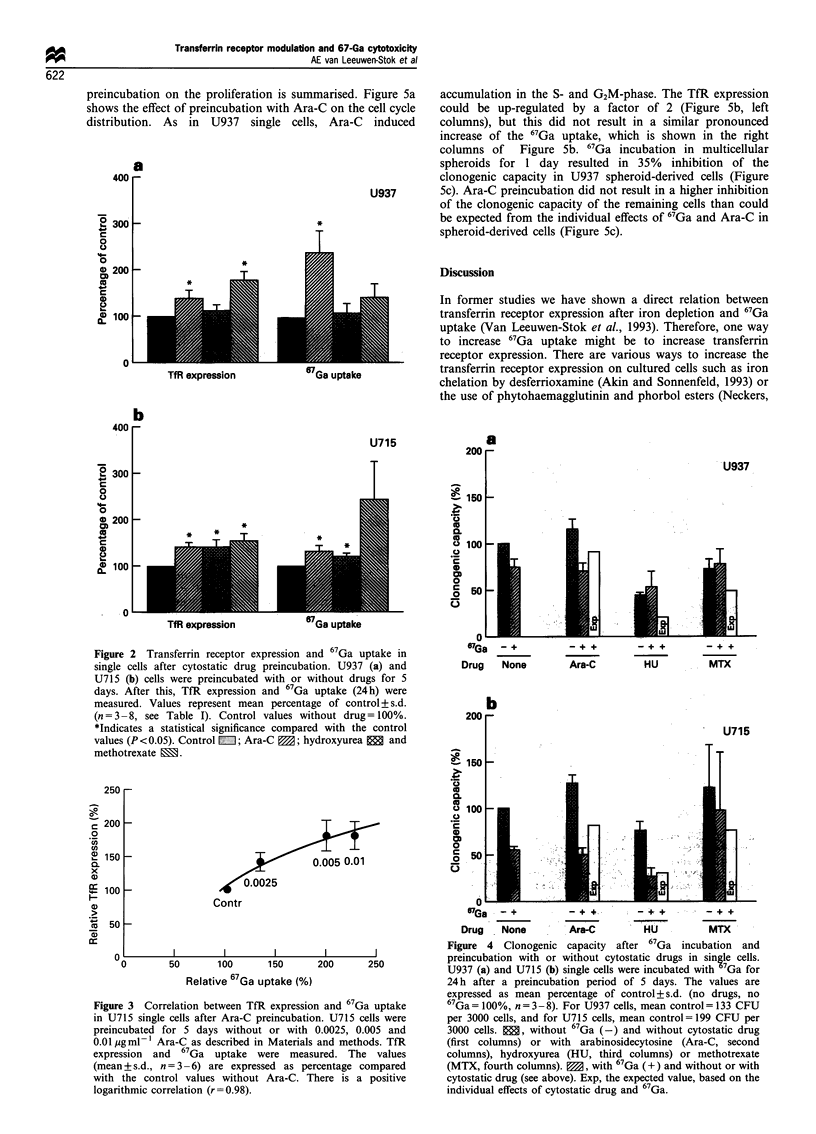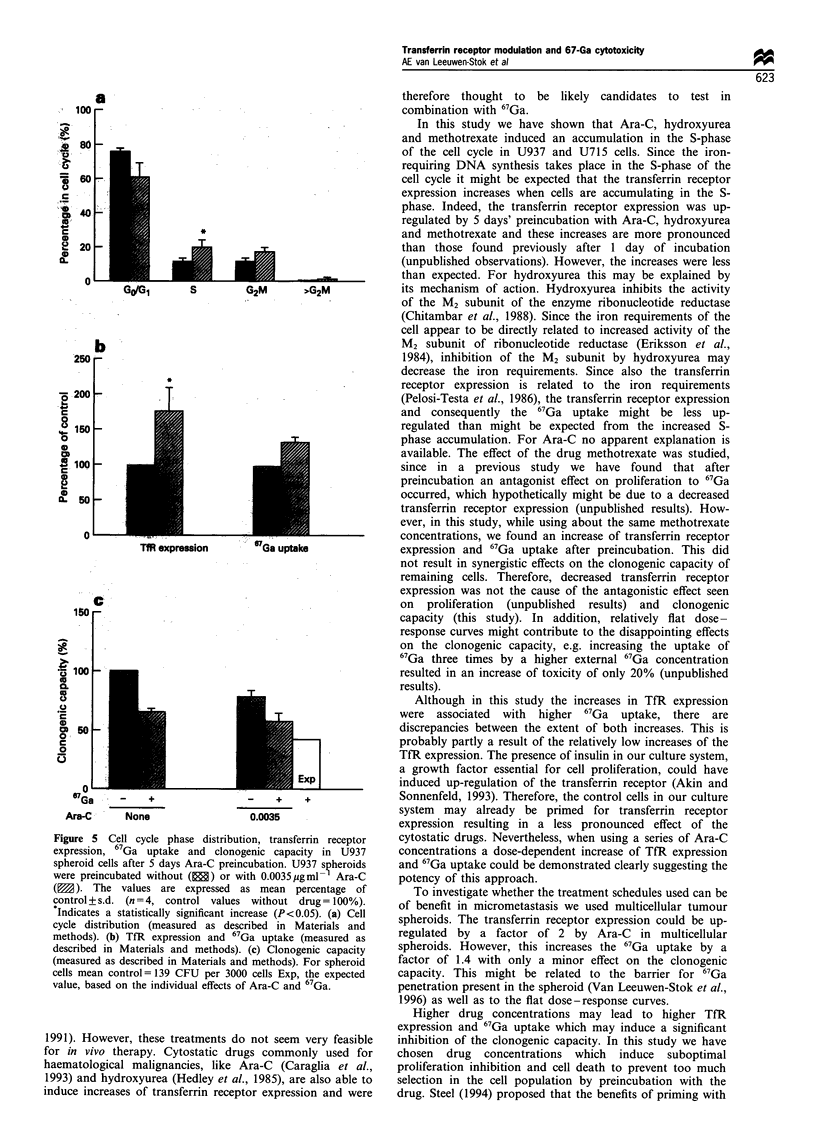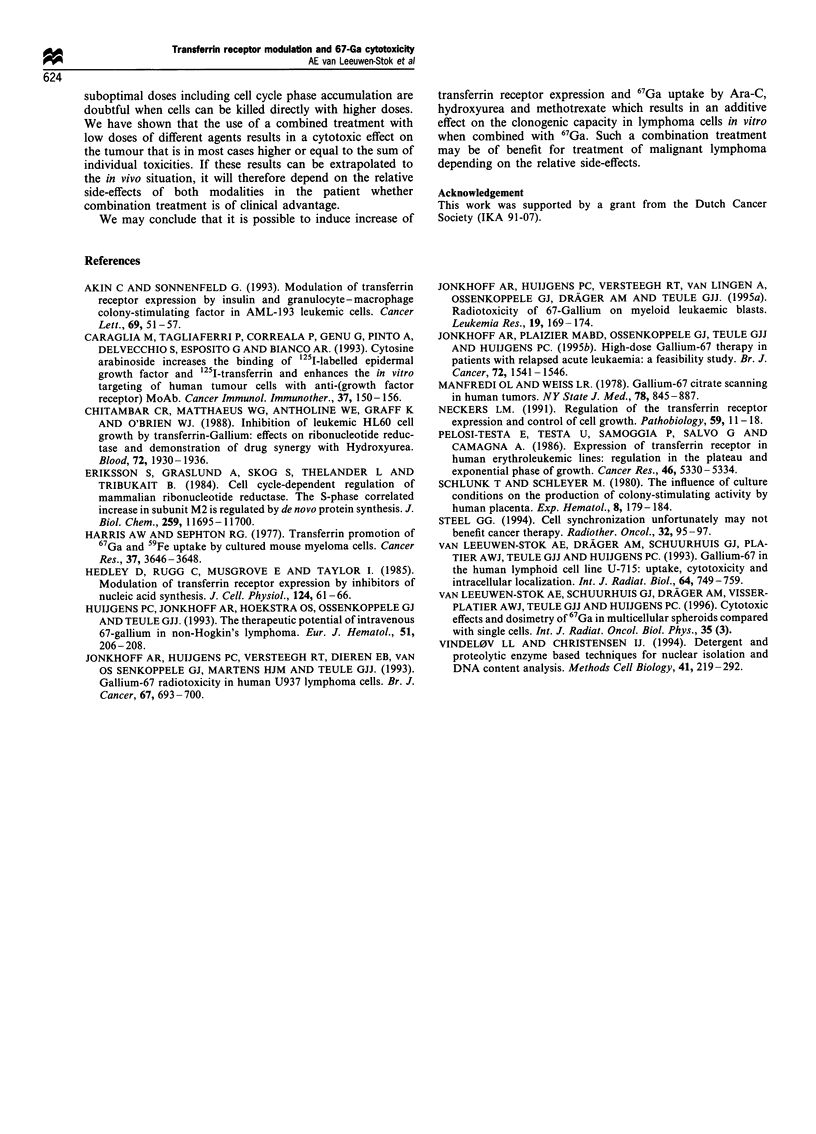# Effect of modulation of the transferrin receptor on gallium-67 uptake and cytotoxicity in lymphoma cell lines.

**DOI:** 10.1038/bjc.1996.411

**Published:** 1996-08

**Authors:** A. E. van Leeuwen-Stok, G. J. Schuurhuis, A. M. Dräger, A. W. Visser-Platier, G. J. Teule, P. C. Huijgens

**Affiliations:** Department of Hematology, Free University Hospital, Amsterdam, The Netherlands.

## Abstract

Gallium-67 is a radionuclide that accumulates in haematological malignancies and is used for diagnostic purposes. Uptake of 67Ga into the cell occurs via the transferrin receptor, which is differentially expressed during the various cell cycle phases. With the aim of selectively increasing 67Ga uptake, we studied whether the transferrin receptor (TfR) expression could be modulated in the U937 and U715 lymphoma cell lines by cytostatic drugs inducing cell cycle phase accumulation. We tested clinically relevant drugs such as 1-beta-D-arabinofuranosylcytosine (Ara-C), hydroxyurea and methotrexate. Cytotoxicity was determined by testing the clonogenic capacity of the lymphoma cell lines. All three drugs induced an increase in S-phase content, TfR expression and 67Ga uptake in U937 and U715 single cells. The combinations of drugs and 67Ga resulted in an additive effect on the clonogenic capacity. In U937 spheroids, cultured by the fibrin clot technique, we found an accumulation in the S-phase too as well as an increase of the transferrin receptor expression after Ara-C preincubation. As in single cells 67Ga uptake was increased without synergistic effects on the clonogenic capacity. In conclusion, priming with drugs induces increased transferrin receptor expression and 67Ga uptake. Inhibition of clonogenic capacity was additive rather than synergistic.


British Journal of Cancer (1996) 74, 619-624
© 1996 Stockton Press All rights reserved 0007-0920/96 $12.00

### Effect of modulation of the transferrin receptor on gallium-67 uptake and cytotoxicity in lymphoma cell lines

AE van Leeuwen-Stok[1], GJ Schuurhuis[1], AM Dräger[1], AWJ Visser-Platier[1], GJJ Teule[2] and PC Huijgens[1]

*Departments of [1]Hematology and [2]Nuclear Medicine, Free University Hospital, Amsterdam, The Netherlands.*

**Summary** Gallium-67 is a radionuclide that accumulates in haematological malignancies and is used for diagnostic purposes. Uptake of $^{67}$Ga into the cell occurs via the transferrin receptor, which is differentially expressed during the various cell cycle phases. With the aim of selectively increasing $^{67}$Ga uptake, we studied whether the transferrin receptor (TfR) expression could be modulated in the U937 and U715 lymphoma cell lines by cytostatic drugs inducing cell cycle phase accumulation. We tested clinically relevant drugs such as 1-$\beta$-D-arabinofuranosylcytosine (Ara-C), hydroxyurea and methotrexate. Cytotoxicity was determined by testing the clonogenic capacity of the lymphoma cell lines. All three drugs induced an increase in S-phase content, TfR expression and $^{67}$Ga uptake in U937 and U715 single cells. The combinations of drugs and $^{67}$Ga resulted in an additive effect on the clonogenic capacity. In U937 spheroids, cultured by the fibrin clot technique, we found an accumulation in the S-phase too as well as an increase of the transferrin receptor expression after Ara-C preincubation. As in single cells $^{67}$Ga uptake was increased without synergistic effects on the clonogenic capacity. In conclusion, priming with drugs induces increased transferrin receptor expression and $^{67}$Ga uptake. Inhibition of clonogenic capacity was additive rather than synergistic.

**Keywords:** gallium-67; transferrin receptor; cytostatic drug; cytoxicity; lymphoma

Gallium-67 citrate ($^{67}$Ga) is an established diagnostic tracer which selectively accumulates in malignancies such as melanoma, lung cancer and malignant lymphoma (Manfredi and Weiss, 1978). Based on the particular emission spectrum of $^{67}$Ga (photons and low-energy electrons) the possibility has been considered that this radionuclide might have a therapeutic potential as well. In previous studies we have described the uptake and radiotoxicity of $^{67}$Ga in haematological cell lines *in vitro* (Jonkhoff *et al.*, 1993; Van Leeuwen-Stok *et al.*, 1993) and in leukaemic blast cells *ex vivo* (Jonkhoff *et al.*, 1995*a*). Furthermore, phase I/II trials with $^{67}$Ga in patients with acute leukaemia and lymphoma were performed and some short-lived responses have been noted (Huijgens *et al.*, 1993; Jonkhoff *et al.*, 1995*b*). Like iron, $^{67}$Ga binds to the transport molecule transferrin and enters the cells via the transferrin receptor (Harris and Sephton, 1977; Van Leeuwen-Stok *et al.*, 1993). The iron requirements of the cell and the expression of the transferrin receptor varies during the cell cycle, being highest during the S- and $G_2$M-phases (E Pelosi-Testa *et al.*, 1986, unpublished observations). Previously, while using iron depletion, we have shown a positive relation between the transferrin receptor expression and $^{67}$Ga uptake in the human lymphoid cell line U715 (Van Leeuwen-Stok *et al.*, 1993). Therefore, increasing $^{67}$Ga uptake in cells may be affected not only by increasing the $^{67}$Ga concentration in the medium surrounding the cells but, more selectively, by increasing the transferrin receptor expression.

Various agents are able to increase transferrin receptor expression, among which are desferrioxamine (Hedley *et al.*, 1985), phorbol esters (Neckers, 1991) and anti-cancer drugs. For example, incubation with 1-$\beta$-D-arabinofuranosylcytosine (Ara-C) induces an increase in the number of transferrin receptors on oropharyngeal carcinoma KB cells (Caraglia *et al.*, 1993). Furthermore, hydroxyurea treatment increased transferrin receptor expression in human T-cell leukaemia cells CCRF-CEM (Hedley *et al.*, 1985).

In this *in vitro* study we examined the effects of Ara-C, hydroxyurea and methotrexate on the expression of the transferrin receptor, $^{67}$Ga uptake and cytotoxicity in two lymphoma cell lines (U937 and U715). In this study both single cells and multicellular spheroids, as a model for micrometastasis (Van Leeuwen-Stok *et al.*, 1996), were used.

Correspondence: AE van Leeuwen-Stok, Free University Hospital, Department of Hematology, BR238, De Boelelaan 1117, 1081 HV Amsterdam, The Netherlands
Received 19 December 1995; revised 30 April 1996; accepted 1 May 1996

### Materials and methods

### *Cells and culture conditions*

U937, a human monoblastic/lymphoid cell line, was obtained commercially (ATCC, Rockville, MD, USA). U715, a human B-cell lymphoid cell line, was generously provided by Dr K Nilsson (University Hospital, Uppsala, Sweden). Cells were maintained in serum-free medium RPMI-1640-L-glutamine (Gibco, Breda, The Netherlands) as described earlier (Van Leeuwen-Stok *et al.*, 1993) containing 25 nM ferrous chloride (Merck, Darmstadt, Germany) and 25 $\mu$g ml$^{-1}$ iron-free purified human transferrin (hTf; Behringwerke, Marburg, Germany). At the start of all experiments the cells were >95% viable as determined by the trypan blue exclusion test. Cell cultures were checked regularly to be negative for mycoplasma using a Gen Probe Kit (Lab Serv Benelux, Apeldoorn, The Netherlands).

### *Multicellular spheroids*

Multicellular spheroids were cultured as described earlier (Van Leeuwen-Stok *et al.*, 1996). In short, $0.2 \times 10^6$ cells were harvested and centrifuged at 2000 *g* for 2 min after which the supernatant was discarded. The cells were resuspended in 7.5 $\mu$l fibrinogen (20 mg ml$^{-1}$; F 4753, Sigma, St Louis, MO, USA) and 4 $\mu$l thrombin (20 U ml$^{-1}$, Merck) solution and incubated at 37°C for 10 min during which a fibrin clot was formed. The cell clots were cultured for 2 days in a spinner flask (Bellco, Vineland, NJ, USA) in a 5% carbon dioxide incubator at 37°C with the magnetic stirrer rotating at 100 r.p.m. Spheroids with a diameter of 2-3 mm were used for experiments. Spheroids were harvested and plated in 24-well plates which were coated with 1% agarose in phosphate-buffered saline (PBS) (Agarose L, Pharmacia, Uppsala, Sweden) to prevent attachment to the bottom of the wells. After drug and/or $^{67}$Ga incubation, spheroids were dispersed into single cells by incubation for 20 min at 37°C with 20 $\mu$l (0.5 U) human plasmin (Chromogenix, Mölndal, Sweden) per spheroid.

*$^{67}$Ga citrate*

$^{67}$Gallium was obtained commerically (Mallinckrodt Diagnostics Holland, Petten, The Netherlands) as $^{67}$Ga chloride. $^{67}$Ga citrate with a low concentration of citrate was prepared as described previously (Van Leeuwen-Stok *et al.*, 1993). The specific activity of the resulting $^{67}$Ga citrate solution was about 40 $\mu$Ci pmol$^{-1}$ $^{67}$Ga. The $^{67}$Ga concentration used in the experiments was 80 $\mu$Ci ml$^{-1}$.

*Cytostatic drugs*

1-$\beta$-D-arabinofuranosylcytosine (Ara-C, 20 mg ml$^{-1}$) was obtained from Multipharma (Weesp, The Netherlands), hydoxyurea (20 mg ml$^{-1}$) from Sigma (St Louis, USA) and methotrexate (25 mg ml$^{-1}$) from Pharmachemie (Haarlem, The Netherlands).

*Preincubation with drugs*

U937 and U715 single cells were preincubated in 25 cm$^3$ culture flasks for 3, 4, 5 and 6 days with various concentrations of the drugs in order to find the optimal preincubation for TfR expression. Drug concentrations are desired which cause an increase of TfR expression but do not extensively inhibit cell proliferation. Fifty per cent inhibition was chosen as the arbitrary maximal inhibiting effect of a single agent so that the sum of the individual effects of drug and $^{67}$Ga could not exceed 100%. The initial cell concentration was $0.2 \times 10^6$ cells ml$^{-1}$ for U937 cells and $0.1 \times 10^6$ cells ml$^{-1}$ for U715 cells. After preincubation the cells were harvested, analysed for TfR expression and DNA distribution and used for $^{67}$Ga uptake and cytotoxicity studies. Multicellular spheroids were preincubated with or without drugs in 24-well plates. After preincubation one part of the spheroids for each of the treatments was dispersed to single cells with plasmin for DNA and TfR analysis. The other part of the spheroids for each of the treatments was washed and plated in fresh medium with or without 80 $\mu$Ci ml$^{-1}$ $^{67}$Ga for 24 h. After this incubation all spheroids were dispersed into single cells for plating in the clonogenic assay.

*Flow cytometric DNA analysis*

Nuclei were prepared for flow cytometric DNA analysis with propidium iodide (PI) according to the method of Vindeløv and Christensen (1994). Nuclei (20 000 events) were analysed using the FACScan (Becton Dickinson, Mountain View, CA). Cell cycle analysis was performed using CellFit DNA analysis software (Becton Dickinson).

*Flow cytometric transferrin receptor analysis*

Transferrin receptor expression was measured using flow cytometry. The anti-TfR monoclonal antibody (MAb; CD71) used was an IgG1 fluorescein isothiocyanate (FITC)-conjugated mouse anti-human MAb. FITC-conjugated mouse IgG1 MAb against F(ab) was used as negative control (Dako, Glostrup, Denmark). Cells were suspended in PBS with 0.1% bovine serum albumin (BSA) in a concentration of $4 \times 10^6$ cells ml$^{-1}$. The MAbs were diluted 20 times with PBS–BSA. Cell suspension (50 $\mu$l) was incubated with 50 $\mu$l MAb for 30 min on ice. Cells were washed twice with PBS–BSA and finally suspended in 500 $\mu$l PBS–BSA. Fluorescence intensity was analysed with the FACScan. Data are expressed as the fluorescence index (FI; arbitrary units) which was calculated as follows:

$$\mathrm{FI} = \frac{\text{MFI sample} - \text{MFI negative control}}{\text{MFI negative control}}$$

where MFI is the mean fluorescence intensity.

*Cellular uptake of $^{67}$Ga*

After the 24 h incubation period spheroids and single cells were transferred to tubes on ice, washed twice with ice-cold PBS and counted in a gamma counter (1470 Wizard, Wallac, Turku, Finland). Hereafter, these spheroids were dispersed, the cells were washed once and counted to distinguish between $^{67}$Ga uptake in the entire spheroid and $^{67}$Ga uptake in the spheroid cells alone. $^{67}$Ga uptake was expressed as follows:

$$\text{Counts per cell} = \frac{\text{c.p.m. pellet} - \text{c.p.m. background}}{\text{cell number}}$$

where c.p.m. is counts per minute, corrected for the half-life of $^{67}$Ga.

*Clonogenic cell survival*

Cells were washed once with PBS and $0.3 \times 10^4$ cells were resuspended in placenta-conditioned Iscove's modified Dulbecco's medium with fetal bovine serum (13%; Gibco), methylcellulose (1%; Fluka Biochemika, Buchs, Germany), glutathione-reductase (100 $\mu$g ml$^{-1}$; Boehringer, Mannheim, Germany), BSA (1%) and human transferrin (0.6 $\mu$g ml$^{-1}$; Behringwerke, Marburg, Germany) according to Schlunk and Schleyer (1980). Cells were plated in 24-well plates (200 $\mu$l per well) and after 7 days of incubation colonies (>40 cells) and clusters (8–40 cells) were counted.

*Statistics*

All data are presented as the mean of *n* experiments (*n* is indicated in Table I). Bars indicate the standard error of the mean. In Figures 1–4, an independent Student's *t* test ($\alpha = 0.05$) has been used. The hypothesis tested was: the difference in means = 0 (no statistically significant difference).

### Results

*Preincubation with cytostatic drugs*

For most concentrations of drugs tested, 5 days of preincubation was optimal for up-regulation of the TfR. The concentrations found to inhibit cell proliferation by 50% at maximum are shown in Table I. It should be noted that these concentrations do not necessarily cause a maximal increase of the TfR expression. For further experiments drug concentrations marked with an asterisk in Table I were used, unless otherwise indicated.

*Cell cycle effect of drug preincubation*

In Figure 1 the effect of 5 days' preincubation on cell cycle distribution in the two cell lines is shown. Preincubation with Ara-C, hydroxyurea and methotrexate induced increases in S-phase cells in both U937 and U715 cells, which represent statistically significant differences from control experiments for all drugs used except for the combination U715 and methotrexate (Figure 1). Furthermore, in U937 cells a tendency towards an increase in the percentage of $G_2M$-phase cells was seen, which was significant for hydroxyurea.

*Transferrin receptor expression and $^{67}$Ga uptake*

All drugs caused an increase of the TfR expression (FI) after 5 days of preincubation in both cell lines, although a statistically significant difference was not reached in all cases (Figure 2a and b). These increased TfR expressions were in qualitative concordance with the $^{67}$Ga uptake shown in the right columns of Figure 2. Here also a statistically significant difference was not reached for all cases. Although increases of the TfR expression and $^{67}$Ga uptake parallel each other, there are discrepancies between the extent of both. This is probably

**Table I** Effect of 5 days' preincubation with cytostatic drugs on the proliferation of U937 and U715 single cells and U937 spheroids

| | *Drug concentration* ($\mu g\,ml^{-1}$) | n | *Proliferation* (%) |
|---|---|---|---|
| U937 single cells | | | |
| Control | – | 8 | 0.82±0.10[a] (100)[b] |
| Ara-C | 0.007[c] | 8 | 0.54±0.15 (66) |
| HU | 5[c] | 5 | 0.45±0.21 (55) |
| MTX | 0.009[c] | 5 | 0.41±0.09 (50) |
| U715 single cells | | | |
| Control | – | 6 | 0.53±0.14 (100) |
| Ara-C | 0.0025[c] | 6 | 0.28±0.10 (53) |
| Ara-C | 0.005 | 3 | 0.23±0.06 (43) |
| Ara-C | 0.01 | 3 | 0.08±0.08 (15) |
| HU | 4[c] | 3 | 0.30±0.09 (57) |
| MTX | 0.0075[c] | 6 | 0.26±0.23 (49) |
| U937 spheroids | | | |
| Control | – | 4 | 0.99±0.21 (100) |
| Ara-C | 0.0035[c] | 4 | 0.68±0.07 (69) |

Proliferation, number of viable cells at the end of the incubation time minus number of viable cells seeded. Initial cell concentration was $0.2 \times 10^6$ cells $ml^{-1}$ for U937, $0.1 \times 10^6$ cells $ml^{-1}$ for U715 cells and $0.2 \times 10^6$ U937 cells per spheroid. [a] Mean ± s.d. × $10^6$ cells $ml^{-1}$. [b] Percentage proliferation compared with control cells. [c] Indicates drug concentation used for further experiments. *n*, number of experiments; Ara-C, 1-*β*-D arabinofuranosyl-cytosine; HU, hydroxyurea; MTX, methotrexate.

**Figure 1** Cell cycle phase distribution of single cells after preincubation with cytostatic drugs. Two cell lines U937 (**a**) and U715 (**b**) were preincubated with or without drugs for 5 days and thereafter assayed for cell cycle accumulation as described in Materials and methods. Values are mean±s.d. ($n = 3-8$, see Table I). *Indicates a statistically significant increase ($P < 0.05$). Control ; Ara-C ; hydroxyurea and methotrexate .

due to the relatively small increase of TfR expression and $^{67}$Ga uptake which may become clear from Figure 3. Figure 3 shows that further increase of the Ara-C concentrations in U715 cells up to cytotoxicity levels that exceeded 50% proliferation inhibition (0.005 and 0.01 $\mu g\,ml^{-1}$) led to a further increase of transferrin receptor expression, which resulted in a relatively large increase of $^{67}$Ga uptake in these cells. Thus, there is a good parallel between increase of TfR expression and $^{67}$Ga uptake but these are relatively small at the drug concentrations that can be used without causing extreme proliferation inhibition.

### *Clonogenic capacity*

Figure 4 shows the clonogenic capacity after $^{67}$Ga treatment in cells preincubated with or without drugs. The open bars indicate the expected clonogenic capacity (Exp), which represents the sum of the individual effects of $^{67}$Ga and drug. This figure shows that $^{67}$Ga incubation after Ara-C preincubation in both cell lines resulted in a higher decrease of the clonogenic capacity than expected (open bars in the second columns). However, the clonogenic capacity than expected (open bars in the second columns). However, the clonogenic capacity after $^{67}$Ga incubation both with or without Ara-C preincubation is equal (compare hatched bars in first and second columns). This may be caused by stimulation of the clonogenic capacity of the residual cells by Ara-C alone, although this stimulation is not statistically significant from the control cells. Since this stimulation by Ara-C itself makes the results difficult to interpret, we also used a higher concentration of Ara-C despite the pronounced proliferation inhibition. In U715 cells 0.01 $\mu g\,ml^{-1}$ Ara-C led to an inhibition of the clonogenic capacity of 50%. When $^{67}$Ga and Ara-C were used together an 86% inhibition was found (data not shown). The expected value of the combination would be 95% inhibition (50% for Ara-C and 45% for $^{67}$Ga) showing not more than an additive effect. $^{67}$Ga incubation after hydroxyurea and methotrexate preincubation resulted in a lower or equal clonogenic capacity compared with the expected values (third and fourth columns of Figure 4).

### *Effect of Ara-C preincubation in multicellular spheroids*

In order to see whether drug preincubation can be of benefit for $^{67}$Ga treatment in a micrometastasis model, we used multicellular spheroids (Van Leeuwen-Stok *et al.*, 1996). Since in the single cell experiments similar effects were found with the three drugs in both cell lines, we have chosen to use one particular combination, Ara-C in U937 spheroids. In Table I the Ara-C concentration used and the effect of preincubation on the proliferation is summarised. Figure 5a shows the effect of preincubation with Ara-C on the cell cycle distribution. As in U937 single cells, Ara-C induced accumulation in the S- and $G_2M$-phase. The TfR expression could be up-regulated by a factor of 2 (Figure 5b, left columns), but this did not result in a similar pronounced increase of the $^{67}$Ga uptake, which is shown in the right columns of Figure 5b. $^{67}$Ga incubation in multicellular spheroids for 1 day resulted in 35% inhibition of the clonogenic capacity in U937 spheroid-derived cells (Figure 5c). Ara-C preincubation did not result in a higher inhibition of the clonogenic capacity of the remaining cells than could be expected from the individual effects of $^{67}$Ga and Ara-C in spheroid-derived cells (Figure 5c).

**Figure 2** Transferrin receptor expression and $^{67}$Ga uptake in single cells after cytostatic drug preincubation. U937 (**a**) and U715 (**b**) cells were preincubated with or without drugs for 5 days. After this, TfR expression and $^{67}$Ga uptake (24 h) were measured. Values represent mean percentage of control ± s.d. ($n = 3-8$, see Table I). Control values without drug = 100%. *Indicates a statistical significance compared with the control values ($P < 0.05$). Control ; Ara-C ; hydroxyurea and methotrexate .

**Figure 3** Correlation between TfR expression and $^{67}$Ga uptake in U715 single cells after Ara-C preincubation. U715 cells were preincubated for 5 days without or with 0.0025, 0.005 and 0.01 $\mu g\,ml^{-1}$ Ara-C as described in Materials and methods. TfR expression and $^{67}$Ga uptake were measured. The values (mean ± s.d., $n = 3-6$) are expressed as percentage compared with the control values without Ara-C. There is a positive logarithmic correlation ($r = 0.98$).

### Discussion

In former studies we have shown a direct relation between transferrin receptor expression after iron depletion and $^{67}$Ga uptake (Van Leeuwen-Stok *et al.*, 1993). Therefore, one way to increase $^{67}$Ga uptake might be to increase transferrin receptor expression. There are various ways to increase the transferrin receptor expression on cultured cells such as iron chelation by desferrioxamine (Akin and Sonnenfeld, 1993) or the use of phytohaemagglutinin and phorbol esters (Neckers,

**Figure 4** Clonogenic capacity after $^{67}$Ga incubation and preincubation with or without cytostatic drugs in single cells. U937 (**a**) and U715 (**b**) single cells were incubated with $^{67}$Ga for 24 h after a preincubation period of 5 days. The values are expressed as mean percentage of control ± s.d. (no drugs, no $^{67}$Ga = 100%, $n = 3-8$). For U937 cells, mean control = 133 CFU per 3000 cells, and for U715 cells, mean control = 199 CFU per 3000 cells. , without $^{67}$Ga (−) and without cytostatic drug (first columns) or with arabinosidecytosine (Ara-C, second columns), hydroxyurea (HU, third columns) or methotrexate (MTX, fourth columns). , with $^{67}$Ga (+) and without or with cytostatic drug (see above). Exp, the expected value, based on the individual effects of cytostatic drug and $^{67}$Ga.

**Figure 5** Cell cycle phase distribution, transferrin receptor expression, $^{67}$Ga uptake and clonogenic capacity in U937 spheroid cells after 5 days Ara-C preincubation. U937 spheroids were preincubated without (▩) or with 0.0035 $\mu$g ml$^{-1}$ Ara-C (▨). The values are expressed as mean percentage of control ± s.d. ($n=4$, control values without drug = 100%). *Indicates a statistically significant increase ($P<0.05$). (**a**) Cell cycle distribution (measured as described in Materials and methods). (**b**) TfR expression and $^{67}$Ga uptake (measured as described in Materials and methods). (**c**) Clonogenic capacity (measured as described in Materials and methods). For spheroid cells mean control = 139 CFU per 3000 cells Exp, the expected value, based on the individual effects of Ara-C and $^{67}$Ga.

1991). However, these treatments do not seem very feasible for *in vivo* therapy. Cytostatic drugs commonly used for haematological malignancies, like Ara-C (Caraglia *et al.*, 1993) and hydroxyurea (Hedley *et al.*, 1985), are also able to induce increases of transferrin receptor expression and were therefore thought to be likely candidates to test in combination with $^{67}$Ga.

In this study we have shown that Ara-C, hydroxyurea and methotrexate induced an accumulation in the S-phase of the cell cycle in U937 and U715 cells. Since the iron-requiring DNA synthesis takes place in the S-phase of the cell cycle it might be expected that the transferrin receptor expression increases when cells are accumulating in the S-phase. Indeed, the transferrin receptor expression was up-regulated by 5 days' preincubation with Ara-C, hydroxyurea and methotrexate and these increases are more pronounced than those found previously after 1 day of incubation (unpublished observations). However, the increases were less than expected. For hydroxyurea this may be explained by its mechanism of action. Hydroxyurea inhibits the activity of the $M_2$ subunit of the enzyme ribonucleotide reductase (Chitambar *et al.*, 1988). Since the iron requirements of the cell appear to be directly related to increased activity of the $M_2$ subunit of ribonucleotide reductase (Eriksson *et al.*, 1984), inhibition of the $M_2$ subunit by hydroxyurea may decrease the iron requirements. Since also the transferrin receptor expression is related to the iron requirements (Pelosi-Testa *et al.*, 1986), the transferrin receptor expression and consequently the $^{67}$Ga uptake might be less up-regulated than might be expected from the increased S-phase accumulation. For Ara-C no apparent explanation is available. The effect of the drug methotrexate was studied, since in a previous study we have found that after preincubation an antagonist effect on proliferation to $^{67}$Ga occurred, which hypothetically might be due to a decreased transferrin receptor expression (unpublished results). However, in this study, while using about the same methotrexate concentrations, we found an increase of transferrin receptor expression and $^{67}$Ga uptake after preincubation. This did not result in synergistic effects on the clonogenic capacity of remaining cells. Therefore, decreased transferrin receptor expression was not the cause of the antagonistic effect seen on proliferation (unpublished results) and clonogenic capacity (this study). In addition, relatively flat dose–response curves might contribute to the disappointing effects on the clonogenic capacity, e.g. increasing the uptake of $^{67}$Ga three times by a higher external $^{67}$Ga concentration resulted in an increase of toxicity of only 20% (unpublished results).

Although in this study the increases in TfR expression were associated with higher $^{67}$Ga uptake, there are discrepancies between the extent of both increases. This is probably partly a result of the relatively low increases of the TfR expression. The presence of insulin in our culture system, a growth factor essential for cell proliferation, could have induced up-regulation of the transferrin receptor (Akin and Sonnenfeld, 1993). Therefore, the control cells in our culture system may already be primed for transferrin receptor expression resulting in a less pronounced effect of the cytostatic drugs. Nevertheless, when using a series of Ara-C concentrations a dose-dependent increase of TfR expression and $^{67}$Ga uptake could be demonstrated clearly suggesting the potency of this approach.

To investigate whether the treatment schedules used can be of benefit in micrometastasis we used multicellular tumour spheroids. The transferrin receptor expression could be up-regulated by a factor of 2 by Ara-C in multicellular spheroids. However, this increases the $^{67}$Ga uptake by a factor of 1.4 with only a minor effect on the clonogenic capacity. This might be related to the barrier for $^{67}$Ga penetration present in the spheroid (Van Leeuwen-Stok *et al.*, 1996) as well as to the flat dose–response curves.

Higher drug concentrations may lead to higher TfR expression and $^{67}$Ga uptake which may induce a significant inhibition of the clonogenic capacity. In this study we have chosen drug concentrations which induce suboptimal proliferation inhibition and cell death to prevent too much selection in the cell population by preincubation with the drug. Steel (1994) proposed that the benefits of priming with suboptimal doses including cell cycle phase accumulation are doubtful when cells can be killed directly with higher doses. We have shown that the use of a combined treatment with low doses of different agents results in a cytotoxic effect on the tumour that is in most cases higher or equal to the sum of individual toxicities. If these results can be extrapolated to the *in vivo* situation, it will therefore depend on the relative side-effects of both modalities in the patient whether combination treatment is of clinical advantage.

We may conclude that it is possible to induce increase of transferrin receptor expression and $^{67}$Ga uptake by Ara-C, hydroxyurea and methotrexate which results in an additive effect on the clonogenic capacity in lymphoma cells *in vitro* when combined with $^{67}$Ga. Such a combination treatment may be of benefit for treatment of malignant lymphoma depending on the relative side-effects.

**Acknowledgement**
This work was supported by a grant from the Dutch Cancer Society (IKA 91-07).

### References

AKIN C AND SONNENFELD G. (1993). Modulation of transferrin receptor expression by insulin and granulocyte–macrophage colony-stimulating factor in AML-193 leukemic cells. *Cancer Lett.*, **69**, 51–57.

CARAGLIA M, TAGLIAFERRI P, CORREALA P, GENU G, PINTO A, DELVECCHIO S, ESPOSITO G AND BIANCO AR. (1993). Cytosine arabinoside increases the binding of $^{125}$I-labelled epidermal growth factor and $^{125}$I-transferrin and enhances the *in vitro* targeting of human tumour cells with anti-(growth factor receptor) MoAb. *Cancer Immunol. Immunother.*, **37**, 150–156.

CHITAMBAR CR, MATTHAEUS WG, ANTHOLINE WE, GRAFF K AND O'BRIEN WJ. (1988). Inhibition of leukemic HL60 cell growth by transferrin-Gallium: effects on ribonucleotide reductase and demonstration of drug synergy with Hydroxyurea. *Blood*, **72**, 1930–1936.

ERIKSSON S, GRASLUND A, SKOG S, THELANDER L AND TRIBUKAIT B. (1984). Cell cycle-dependent regulation of mammalian ribonucleotide reductase. The S-phase correlated increase in subunit M2 is regulated by *de novo* protein synthesis. *J. Biol. Chem.*, **259**, 11695–11700.

HARRIS AW AND SEPHTON RG. (1977). Transferrin promotion of $^{67}$Ga and $^{59}$Fe uptake by cultured mouse myeloma cells. *Cancer Res.*, **37**, 3646–3648.

HEDLEY D, RUGG C, MUSGROVE E AND TAYLOR I. (1985). Modulation of transferrin receptor expression by inhibitors of nucleic acid synthesis. *J. Cell. Physiol.*, **124**, 61–66.

HUIJGENS PC, JONKHOFF AR, HOEKSTRA OS, OSSENKOPPELE GJ AND TEULE GJJ. (1993). The therapeutic potential of intravenous 67-gallium in non-Hogkin's lymphoma. *Eur. J. Hematol.*, **51**, 206–208.

JONKHOFF AR, HUIJGENS PC, VERSTEEGH RT, DIEREN EB, VAN OS SENKOPPELE GJ, MARTENS HJM AND TEULE GJJ. (1993). Gallium-67 radiotoxicity in human U937 lymphoma cells. *Br. J. Cancer*, **67**, 693–700.

JONKHOFF AR, HUIJGENS PC, VERSTEEGH RT, VAN LINGEN A, OSSENKOPPELE GJ, DRÄGER AM AND TEULE GJJ. (1995*a*). Radiotoxicity of 67-Gallium on myeloid leukaemic blasts. *Leukemia Res.*, **19**, 169–174.

JONKHOFF AR, PLAIZIER MABD, OSSENKOPPELE GJ, TEULE GJJ AND HUIJGENS PC. (1995*b*). High-dose Gallium-67 therapy in patients with relapsed acute leukaemia: a feasibility study. *Br. J. Cancer*, **72**, 1541–1546.

MANFREDI OL AND WEISS LR. (1978). Gallium-67 citrate scanning in human tumors. *NY State J. Med.*, **78**, 845–887.

NECKERS LM. (1991). Regulation of the transferrin receptor expression and control of cell growth. *Pathobiology*, **59**, 11–18.

PELOSI-TESTA E, TESTA U, SAMOGGIA P, SALVO G AND CAMAGNA A. (1986). Expression of transferrin receptor in human erythroleukemic lines: regulation in the plateau and exponential phase of growth. *Cancer Res.*, **46**, 5330–5334.

SCHLUNK T AND SCHLEYER M. (1980). The influence of culture conditions on the production of colony-stimulating activity by human placenta. *Exp. Hematol.*, **8**, 179–184.

STEEL GG. (1994). Cell synchronization unfortunately may not benefit cancer therapy. *Radiother. Oncol.*, **32**, 95–97.

VAN LEEUWEN-STOK AE, DRÄGER AM, SCHUURHUIS GJ, PLATIER AWJ, TEULE GJJ AND HUIJGENS PC. (1993). Gallium-67 in the human lymphoid cell line U-715: uptake, cytotoxicity and intracellular localization. *Int. J. Radiat. Biol.*, **64**, 749–759.

VAN LEEUWEN-STOK AE, SCHUURHUIS GJ, DRÄGER AM, VISSER-PLATIER AWJ, TEULE GJJ AND HUIJGENS PC. (1996). Cytotoxic effects and dosimetry of $^{67}$Ga in multicellular spheroids compared with single cells. *Int. J. Radiat. Oncol. Biol. Phys.*, **35 (3)**.

VINDELØV LL AND CHRISTENSEN IJ. (1994). Detergent and proteolytic enzyme based techniques for nuclear isolation and DNA content analysis. *Methods Cell Biology*, **41**, 219–292.